# Identification and characterization of *Anopheles* spp. breeding habitats in the Korhogo area in northern Côte d’Ivoire: a study prior to a *Bti*-based larviciding intervention

**DOI:** 10.1186/s13071-019-3404-0

**Published:** 2019-03-27

**Authors:** Barnabas Zogo, Alphonsine A. Koffi, Ludovic P. Ahoua Alou, Florence Fournet, Amal Dahounto, Roch Kounbobr Dabiré, Lamine Baba-Moussa, Nicolas Moiroux, Cédric Pennetier

**Affiliations:** 1 Institut Pierre Richet (IPR), Bouaké, Côte d’Ivoire; 2 MIVEGEC, IRD, CNRS, Univ. Montpellier, Montpellier, France; 3 Faculté des Sciences et Techniques, Université d’Abomey Calavi, Abomey-Calavi, Benin; 4 CEMV, Université Alassane Ouattara, Bouaké, Côte d’Ivoire; 5 Institut de Recherche en Sciences de la Santé (IRSS), Bobo Dioulasso, Burkina Faso

**Keywords:** Malaria, Larvae, Rice, Larviciding, Randomized controlled trial

## Abstract

**Background:**

Although larviciding may be a valuable tool to supplement long-lasting insecticide nets (LLINs) in West Africa in different ecological settings, its actual impact on malaria burden and transmission has yet to be demonstrated. A randomized controlled trial was therefore undertaken to assess the effectiveness of larviciding using *Bacillus thuringiensis israeliensis* (*Bti*) in addition to the use of LLINs. In order to optimally implement such a larviciding intervention, we first aimed to identify and to characterize the breeding habitats of *Anopheles* spp. in the entire study area located in the vicinity of Korhogo in northern Côte d’Ivoire.

**Methods:**

We conducted two surveys during the rainy and the dry season, respectively, in the thirty villages around Korhogo involved in the study. In each survey, water bodies located within a 2 km radius around each village were identified and assessed for the presence of mosquito larvae. We morphologically identified the larvae to the genus level and we characterized all of the habitats positive for *Anopheles* spp. larvae based on a predefined set of criteria.

**Results:**

Overall, 620 and 188 water bodies positive for *Anopheles* spp. larvae were sampled in the rainy and the dry season, respectively. A broad range of habitat types were identified. Rice paddies accounted for 61% and 57% of the habitats encountered in the rainy and the dry season, respectively. In the rainy season, edges of rivers and streams (12%) were the second most abundant habitats for *Anopheles* spp. larvae. More than 90% of the *Anopheles* spp. breeding habitats were surrounded by green areas. Dams, ponds and drains produced higher numbers of *Anopheles* spp. larvae per square meter than rice paddies (RR = 1.51; 95% CI: 1.18–1.94; *P* = 0.0010). The density of *Anopheles* spp. larvae was significantly higher in habitats surrounded by low-density housing (RR = 4.81; 95% CI: 1.84–12.60; *P* = 0.0014) and green areas (RR = 3.96; 95% CI: 1.92–8.16; *P* = 0.0002] than habitats surrounded by high-density housing. Turbid water [RR = 1.42 (95% CI: 1.15–1.76; *P* = 0.0012) was associated with higher densities of *Anopheles* spp. larvae. The likelihood of finding mosquito pupae in *Anopheles* spp. breeding habitats was higher in the dry season (OR = 5.92; 95% CI: 2.11–16.63; *P* = 0.0007) than in the rainy season.

**Conclusions:**

Rice paddies represented the most frequent habitat type for *Anopheles* spp. larvae in the Korhogo area during both the rainy and the dry seasons. *Anopheles* spp. breeding habitats covered a very large and dynamic area in the rainy season whereas they were fewer in number in the dry season. In this context, implementing a larviciding strategy from the end of the rainy season to the dry season is presumably the most cost-effective strategy.

## Background

Following the development of insecticide-based tools such as indoor residual spraying (IRS) and insecticide treated nets (ITN), the focus of malaria vector control has shifted from larval to adult control [1]. These tools that reduce vector survivorship have proved to exhibit a strong impact on vectorial capacity and are accountable for the recent decrease in malaria burden [2, 3]. Nevertheless, with growing concerns of insecticide resistance and a shift to early feeding and outdoor dwelling vectors, the World Health Organization (WHO) has promoted the Integrated Vector Management (IVM) approach. In this approach, multiple control tools are combined to improve their efficacy, cost-effectiveness and sustainability [4]. Accordingly, larval source management (LSM) is now being reappraised in Africa as a supplementary tool for vector control with the objective of targeting *Anopheles* populations including those causing the residual transmission despite high LLIN/IRS coverage [5].

LSM targets the immature stage of mosquitoes, which is more vulnerable to intervention as mosquito larvae are confined to their breeding habitats and are hence unable to avoid being exposed to treatments. This control method has been associated with the malaria eradication successes achieved to date either through modification of larval habitats or through chemical larvicide interventions [6, 7]. In Africa, LSM has so far received scant attention, although the striking success of *Anopheles gambiae* eradication in Brazil by larviciding may now drive its implementation in similar settings in Africa [7]. According to the WHO interim recommendations, LSM is more suitable for urban areas where the number, the type, and the access to larval habitats can allow for adequate coverage [8]. Many studies have provided evidence of the effectiveness of LSM in different settings in Africa, especially in urban areas [5, 9, 10]. Very few studies have been carried out, however, in rural areas of Africa because *Anopheles* spp. habitats are often poorly defined and blanket treatment of all water bodies is clearly technically and financially challenging. It has been shown that when aquatic habitats are too numerous, LSM is likely to fail unless the most productive habitats are targeted and the levels of coverage limited [11]. Interestingly, a model developed by Killeen et al. [12] predicted that ITN combined with larval control that reduces the number of emerging adult vectors by 50% can result in a 15- to 25-fold reduction in the entomological inoculation rate (EIR), even in highly endemic areas. Moreover, a larval control that targets the most productive habitats has been shown to be more cost-effective [11].

Côte d’Ivoire, in West Africa, is among the top 15 countries in the world that account for more than three-quarters of the global malaria burden [3]. Vector control in Côte d’Ivoire, as in most sub-Saharan countries, mainly relies on the use of LLINs. The Korhogo region in northern Côte d’Ivoire is a lowland rice cultivation area where the density of adult vectors has been reported to be very high, especially during the rainy season [13]. It has been hypothesized that rice production may contribute to relieving the burden of malaria by raising living standards, which in turn can make medicines, quality housing and adequate nutrition more affordable [14]. However, rice fields are known to provide suitable breeding habitats for *Anopheles gambiae* (*s.l.*), which is the main malaria vector in sub-Saharan Africa [14].

Unlike adult vector control tools that are implemented almost systematically in homes, *Anopheles* spp. breeding habitats need to be sought out prior to LSM interventions. To date, studies on *Anopheles* spp. larval ecology have been rather limited in Africa, mainly due to the overreliance on adult vector control tools and the difficulty with sampling in areas where larval habitats are numerous and temporary. Historical data on *Anopheles* spp. larval ecology indicate that *An. gambiae* (*s.l.*) larvae develop in freshwater habitats that are small, temporary, clean and sun-exposed [15]. However, there is a growing body of evidence that these larvae may in fact breed in any available water, even in dirty and polluted habitats [1, 16]. In this study, we identified and characterized *Anopheles* spp. breeding habitats in the Korhogo area. Moreover, in order to rank the breeding habitats, we analyzed their productivity and their capacity to allow completion of larval development. We conducted the present study prior to a randomized controlled trial (RCT) in the frame of the project called REACT, which aims to assess the effectiveness of four strategies complementary to ITNs, including larviciding with *Bacillus thuringiensis israeliensis* (*Bti*) in the Korhogo area in northern Côte d’Ivoire. *Bti* has been chosen because it is highly selective and the probability of development of resistance to *Bti* in the field is very low [5].

## Methods

### Study site

**Fig. 1:**
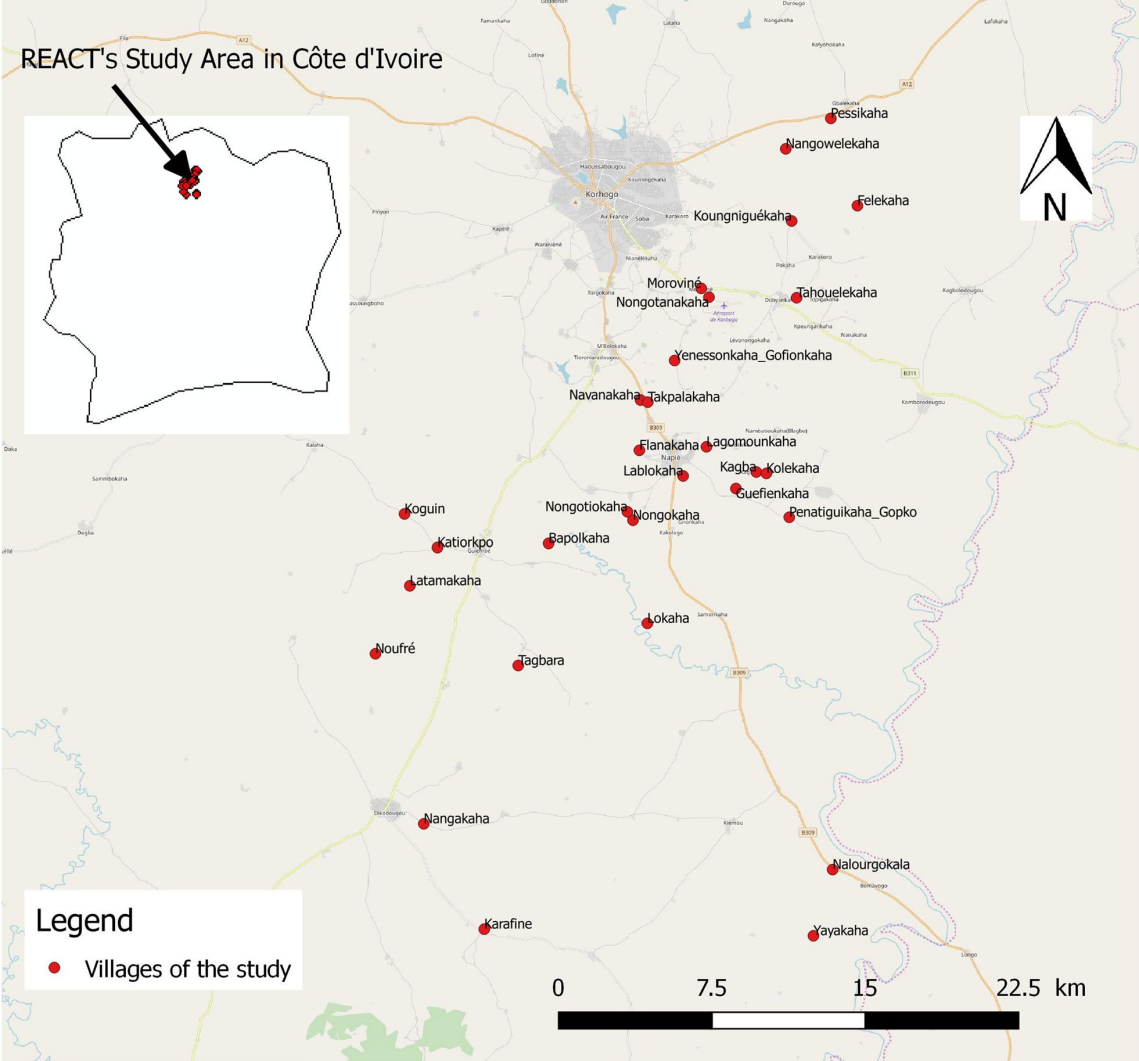
Map of the study area

The study area included 30 villages of the department of Korhogo (9°10′–9°40′N, 5°20′–5°60′W) located in northern Côte d’Ivoire, West Africa (Fig. 1). The villages were selected based on an average population size of 300 inhabitants, a distance between two villages of at least 2 km, and accessibility during the rainy season. The Korhogo department is characterized by a Sudanese climate with a unimodal rainfall regimen from May to November. The annual rainfall varies from 1200 to 1400 mm while the mean annual temperature ranges between 21–35 °C. The minimum temperatures can drop to 16 °C in January due to the Harmattan wind during December and January. The vegetation is a mixture of savannah and open forest characterized by trees and shrubs that are approximately 8–15 m in height. The Korhogo department is fed by tributaries of the Bandama River such as the Naramou and the Solomougou, which dry out considerably in the dry season. Nevertheless, the area has a high density of water dams that allow agriculture to be practiced throughout the year [17]. Therefore, in areas where the soil is highly conducive to agriculture, most of the local inhabitants are farmers and their staple crops include rice, maize and cotton. Rice is mainly cultivated during the rainy season in flooded soils although it is also occasionally planted in the dry season in irrigated areas in the vicinity of dams.

### Larval sampling

Two surveys were conducted from 28th October to 8th November 2016 and from 16th to 27th March 2017 during the rainy and the dry season, respectively. In each of the 30 villages, the field team was made up of one or two experienced technicians from the Institut Pierre Richet (IPR) and two well-trained villagers. Water bodies located within a 2 km radius from each village were identified and subsequently checked for the presence of mosquito larvae. Before sampling, a waiting period of 1–2 min was observed to allow mosquito larvae, if there were any, to rise to the surface. Thereafter, a 350-ml dipper was drawn along the edge of each water body and filled at the end of the stroke according to the standard dipping method [18]. To ascertain the presence or absence of mosquito larvae and to determine the mean larval density per habitat, up to 10 dips were taken at intervals, depending on the size of the water body. The mosquito larvae were morphologically identified to the genus level using reference keys [19]. A given habitat was considered positive when at least one immature *Anopheles* spp. was found. The mosquito larvae were then sorted by genus and counted, and the mean densities of larvae and pupae were recorded.

### Characterization of larval habitats

All of the water bodies positive for the presence of *Anopheles* spp. larvae were georeferenced using a global positioning system (GPS) using Android tablets (Samsung Galaxy version 7.0 Plus). They were visually characterized using a questionnaire that was completed using ODK Collect software. The technicians had been trained together for three sessions to achieve uniformity in the estimations. Then, for each *Anopheles* spp. breeding site, the habitat type (i.e. rice paddy, pond, puddle, agricultural trench, dam, animal/human footprints, basin of water, cesspool, troughs attached to village pumps, irrigation canals and drains, village pumps, or edges of rivers and streams) was identified and the surface area of the breeding site was categorized into one of three groups: (i) small habitat area (< 1 m^2^); (ii) medium habitat area (1–10 m^2^); and (iii) large habitat area (> 10 m^2^). The turbidity of the breeding habitats was classified into one of the following categories: (i) transparent; (ii) turbid, when the bottom of the dipper was still visible; or (iii) very turbid, when the bottom of the dipper was invisible. The distance to the nearest human settlement was estimated and classified into one of three groups: (i) ≤ 100 m; (ii) 101–500 m; and (iii) > 500 m. The general surroundings of each larval habitat were also described as follows: (i) high-density housing with more than 20 houses within a 50 m radius from the habitat; (ii) low-density housing with less than 20 houses within a 50 m radius from the habitat; and (iii) green areas with no houses within a 50 m radius from the habitat. The extent of exposure to sunlight was classified as either (i) sunny, when at least three-quarters of the larval habitat was exposed to sunlight; (ii) shaded, when less than one-quarter of the larval habitat was exposed to sunlight; or (iii) partly-shaded, when more than one-quarter and less than three-quarters of the larval habitat was exposed to sunlight. The crop (rice, maize, cotton, etc.) that surrounded each breeding habitat, the water movement (flowing or stagnant), the density of *Culex* sp. larvae, the density of *Aedes* sp. larvae, the presence of other insects, fish, tadpoles, solid wastes, and liquid wastes were also recorded.

### Data analysis

Statistical analyses were performed using R software [20]. For each variable (habitat type, size, water turbidity, sunlight exposure, distance from houses, general surroundings, vegetation, and cohabitation with *Culex* species), we used a Chi-square test to compare the distribution of *Anopheles* spp. breeding habitats in the different categories between the rainy and the dry season. A Pearson’s Chi-square test with a simulated *P*-value (based on 2000 replicates) was used when conditions did not meet Chi-square test criteria.

We used a zero-truncated negative binomial mixed effect model to analyze the density of *Anopheles* spp. larvae at breeding sites (function ‘glmmadmb’ from the package *glmmADMB*) [21]. The zero-truncated distribution was used as only breeding sites positive for the presence of *Anopheles* spp. larvae were recorded. The village was used as a random intercept in order to account for possible autocorrelation between breeding sites from the same buffer area. The characteristics recorded to describe the breeding sites were used as a fixed effect. We fitted a full model (with all of the characteristics as covariates) and then performed a backward elimination of the less significant covariates according to deviance analysis. Using the same approach, we analyzed the presence (using a binomial model, function ‘glmer’ from package ‘*lme4*’) [22] and the density of pupae (using a negative binomial model, function ‘glmmadmb’).

When the number of breeding sites in a category of predictor was small, this category was pooled with another relevant category of the same predictor. Thus, dams, ponds and puddles were pooled in a common category of size-varying natural waterbodies of habitat types. Agricultural trenches, irrigation canals and drains were pooled in a single category called agriculture-made pools (other than rice paddies). Animal/human footprints, troughs attached to pumps, and village pumps were pooled in a human-made pools category. A new categorical variable describing the presence of other animals (potential predators) living in the breeding sites was created. It was based on the record of the presence of insects, fish, tadpoles, mollusks, etc. that were converted into one of three categories: (i) absence of predators; (ii) presence of invertebrates (insects and/or mollusks); and (iii) presence of invertebrate and/or vertebrates. The categories ‘turbid’ and ‘very turbid’ for the turbidity variable were pooled.

## Results

### Variability of *Anopheles* spp. breeding habitats between the rainy and the dry season

Of the 808 water bodies positive for *Anopheles* spp. larvae sampled during the study period, 620 (77%) were recorded in the rainy season and 188 (23%) in the dry season (Table 1).

**Table 1:** Characterization of *Anopheles* spp. larval habitats in the Korhogo area in the rainy and the dry seasons

Characteristics	Category	No. (%) of breeding habitats for *Anopheles* spp. mosquitoes		Mean no. of *Anopheles* spp. larvae/350 ml		Mean no. of mosquito pupae/350 ml	
		Rainy season	Dry season	Rainy season	Dry season	Rainy season	Dry season
Habitat type	Dam	0 (0)	15 (7.98)	–	13.87	–	0
	Agricultural trench	2 (0.32)	0 (0)	7.50	0	2.50	0
	Basin of water	1 (0.16)	0 (0)	0	0	0	0
	Cesspool	1 (0.16)	0 (0)	0	0	1.00	0
	Edges of rivers and streams	76 (12.26)	4 (2.13)	7.09	3.25	0.18	0
	Animal/human footprints	49 (7.90)	9 (4.79)	7.00	3.67	0.18	0
	Irrigation canals and drains	47 (7.58)	3 (1.60)	11.15	17.67	0.26	0
	Pond	8 (1.29)	0 (0)	19.63	0	0	0
	Puddle	51 (8.23)	50 (26.60)	11.75	12.26	0.47	0.20
	Rice paddy	380 (61.29)	107 (56.91)	7.67	8.40	0.41	0.26
	Troughs attached to village pumps	1 (0.16)	0 (0)	2.00	0	0	0
	Village pumps	4 (0.65)	0 (0)	6.75	0	0.25	0
	Total	620 (100)	188 (100)	8.00	10.00	0	0
Size (m^2^)	< 1	461 (74.35)	130 (69.15)	8.57	8.53	0.42	0.20
	1–10	83 (13.39)	40 (21.28)	8.31	12.90	0.07	0.05
	> 10	76 (12.26)	18 (9.57)	6.37	10.78	0.29	0.56
Water turbidity	Transparent	397 (64.03)	118 (62.77)	6.51	9.15	0.10	0.22
	Turbid	215 (34.68)	69 (36.70)	11.27	10.58	0.85	0.17
	Very turbid	8 (1.29)	1 (0.53)	14.75	9.00	0	0
Distance from houses	< 100 m	26 (4.19)	6 (3.19)	8.46	1.67	1.23	0
	100–500 m	148 (23.87)	61 (32.45)	9.96	7.54	0.99	0.21
	> 500 m	446 (71.94)	121 (64.36)	7.70	11.15	0.09	0.21
Sunlight exposure	Sunny	593 (95.65)	188 (100)	8.45	9.68	0.35	0.20
	Shaded	27 (4.35)	0 (0)	4.19	0	0.44	0.00
	Partly shaded	0 (0)	0 (0)	–	–	–	–
General surroundings	High-density housing	17 (2.74)	2 (1.06)	4.76	0.50	1.41	0
	Low-density housing	10 (1.61)	12 (6.38)	24.80	3.17	0.80	0.17
	Green areas	593 (95.65)	174 (92.55)	8.00	10.23	0	0.21
*Culex* spp. larvae	Present	172 (27.74)	96 (51.06)	7.60	8.05	0.90	0.26
	Absent	448 (72.26)	92 (48.94)	8.52	11.37	0.15	0.14
*Aedes* spp. larvae	Present	0 (0)	5 (2.66)	–	9.8	–	0
	Absent	620 (100)	183 (97.34)	8.27	9.67	0.35	0.21
Other insects	Present	421 (67.90)	152 (80.85)	8.02	10.97	0.17	0.19
	Absent	199 (32.10)	36 (19.15)	8.79	4.19	0.74	0.25
Fish	Present	34 (5.48)	1 (0.53)	8.85	12.00	0.47	0
	Absent	586 (94.52)	187 (99.47)	8.23	9.66	0.35	0.20
Tadpoles	Present	32 (5.16)	16 (8.51)	10.16	11.31	0.44	0
	Absent	588 (94.84)	172 (91.49)	8.16	9.52	0.35	0.22
Crop	Rice	380 (61.29)	107 (56.91)	7.67	8.40	0.41	0.26
	Vegetable gardens	95 (15.32)	52 (27.66)	10.63	13.92	0.20	0.15
	Other	80 (12.90)	586 (15.43)	12.00	8.00	0.32	0.32
Water movement	Flowing	26 (4.19)	3 (1.59)	8.81	3.33	1.27	0
	Stagnant	594 (95.81)	185 (98.40)	8.24	9.78	0.31	0.21
Solid waste	Absent	460 (74.19)	21 (11.17)	8.84	10.81	0.46	0.48
	Present	160 (25.81)	167 (88.83)	6.63	9.53	0.06	0.17
Liquid waste	Absent	593 (95.65)	155 (82.45)	8.32	10.61	0.34	0.21
	Present	27 (4.35)	33 (17.55)	7.22	5.30	0.63	0.18
Vegetation	Absent	21 (3.38)	11 (5.85)	10.00	3.55	0.38	0.09
	Present	599 (96.61)	177 (94.15)	8.21	10.06	0.35	0.21

*Anopheles* spp. breeding habitats were categorized into six types during both the rainy and the dry seasons. Rice paddies (61%), followed by edges of rivers and streams (12%), were the most abundant habitats for *Anopheles* spp. larvae in the rainy season. In the dry season, rice paddies (57%) and puddles (27%) were the most abundant breeding habitats for *Anopheles* spp. larvae (Table 1). The distribution of *Anopheles* spp. breeding sites in the habitat types varied significantly between the dry and the rainy season (*χ*^2^ = 12.43, simulated *P* = 5.10^−4^).

Out of all the *Anopheles* spp. breeding habitats sampled, 461 (74%) and 130 (69%) were small in size (< 1 m^2^) during the rainy and dry season, respectively. Large habitats (> 10 m^2^) accounted for 12 and 10% of the habitats sampled in the rainy and dry season, respectively. Small habitats in rice paddies were the most frequent habitats for *Anopheles* spp. larvae during both the rainy and the dry season (Table 1). Medium-sized habitats (1–10 m^2^) represented 13.39 and 21.28% of the habitats sampled in the rainy and dry season, respectively. The distribution of *Anopheles* spp. breeding habitats in the three different size classes did not vary significantly between the rainy and the dry season (*χ*^2^ = 2.9768, *df* = 2, *P* = 0.2257).

An average of ~ 64% of the *Anopheles* spp. breeding habitats were transparent while ~36% were turbid, irrespective of the season (Table 1). There were no significant differences in the turbidity of the *Anopheles* spp. breeding habitats between the rainy and the dry season (*χ*^2^ = 0.7412, simulated *P* = 0.7231).

The majority (> 95%) of the *Anopheles* spp. breeding habitats were exposed to sunlight, irrespective of the season (Table 1). The proportion of *Anopheles* spp. breeding habitats that were exposed to sunlight was significantly higher in the dry season than in the rainy season (*χ*^2^ = 5.495, *df* = 1, *P* = 0.01907).

*Anopheles* spp. breeding habitats located at a distance > 500 m from houses represented 72% and 64% of the total habitats found in the rainy and the dry season, respectively. Only 4% and 3% of the *Anopheles* spp. breeding habitats were close to houses (i.e. < 100 m) in the rainy and the dry season, respectively (Table 1). There were no significant differences in the distribution of the *Anopheles* spp. breeding habitats in the three classes of distance from human houses between the rainy and the dry season (*χ*^2^ = 2.8104, *df* = 2, *P* = 0.2453).

The majority (> 90%) of the *Anopheles* spp. breeding habitats in both the rainy and the dry seasons were surrounded by green areas, with no houses within a 50 m radius (Table 1). The distribution of *Anopheles* spp. breeding habitats in the three classes of general surroundings did not vary significantly between the rainy and the dry season (*χ*^2^ = 3.1011, simulated *P* = 0.2524).

*Culex* spp. larvae were present in 28% and 51% of the *Anopheles* spp. breeding habitats sampled in the rainy and the dry season, respectively (Table 1). The proportion of *Anopheles* spp. breeding habitats that contained *Culex* spp. larvae was significantly higher in the dry season than the rainy season (*χ*^2^ = 43.72, *df* = 1, *P* < 0.0001).

More than 95% of the *Anopheles* spp. breeding habitats sampled were found in stagnant water, irrespective of the season. The proportion of *Anopheles* spp. breeding habitats in stagnant water did not differ significantly between the rainy and the dry season (*χ*^2^ = 3.4144, simulated *P* = 0.1959).

### Factors determining the density of *Anopheles* spp. larvae

Dams, ponds and drains produced higher numbers of *Anopheles* spp. larvae than rice paddies (RR = 1.51; 95% CI: 1.18–1.94; *P* = 0.0010; Table 2). The density of *Anopheles* spp. larvae was significantly higher in habitats surrounded by low-density housing (RR = 4.81; 95% CI: 1.84–12.60; *P* = 0.0014; Table 2) and green areas (RR = 3.96; 95% CI: 1.92–8.16; *P* = 0.0002; Table 2) than habitats surrounded by high-density housing. Turbid water (RR = 1.42; 95% CI: 1.15–1.76; *P* = 0.0012; Table 3) was associated with a higher density of *Anopheles* spp. larvae (Table 2). Shaded habitats had lower densities of *Anopheles* spp. larvae than sun-exposed habitats (RR = 0.51; 95% CI: 0.31–0.86; *P* = 0.0110; Table 2).

**Table 2:** Multiple regression analysis for factors determining the density of *Anopheles* spp. larvae

Factor	Levels	Rate ratio	95% CI	*P*-value
Habitat type	Rice paddies	1		0.4815
	Irrigation canals and drains	1.17	0.81–1.69	0.4088
	Dams, ponds and puddles	1.51	1.18–1.94	0.0010***
	Edges of rivers and streams	1.02	0.74–1.42	0.8935
	Animal/human footprints	1.43	0.93–2.19	0.1059
	Others	1.47	0.70–3.10	0.3068
General surroundings	High-density housing	1		
	Low-density housing	4.81	1.84–12.60	0.0014**
	Green areas	3.96	1.92–8.16	0.0002***
Water turbidity	Transparent	1		
	Turbid	1.42	1.15–1.76	0.0012**
Sunlight	Sunny	1		
	Shaded	0.51	0.31–0.86	0.0110*

*P < 0.05, **P < 0.01, ***P < 0.001

**Table 3:** Multiple regression analysis for factors determining the presence of mosquito pupae

Factor	Levels	Odds ratio	95% CI	*P*-value
Season	Rainy season	1		
	Dry season	5.92	2.11–16.63	0.0007***
Habitat type	Rice paddies	1		
	Irrigation canals and drains	1.43	0.35–5.82	0.6151
	Dams, ponds and puddles	0.25	0.08–0.77	0.0155*
	Edges of rivers and streams	0.24	0.05–1.17	0.0775
	Animal/human footprints	0.05	0–1.29	0.0704
	Others	0.22	0.01–4.77	0.3376
General surroundings	High-density housing	1		
	Low-density housing	0.31	0.01–7.58	0.4715
	Green area	0.03	0–0.70	0.0296*
Distance from human houses	100–500 m	1		
	< 100 m	0.28	0.02–3.80	0.3406
	> 500 m	0.25	0.10–0.66	0.0050**
Sunlight	Sunny	1		
	Shaded	0.10	0.01–1.18	0.0671

*P < 0.05, **P < 0.01, ***P < 0.001

*Abbreviation*: CI, confidence interval

### Factors determining the presence of mosquito pupae in *Anopheles* breeding habitats

The likelihood of finding mosquito pupae in the *Anopheles* spp. breeding habitats was higher during the dry season (OR = 5.92; 95% CI: 2.11–16.63; *P* = 0.0007; Table 3) than during the rainy season. Dams, ponds and drains were less likely to contain mosquito pupae than rice paddies (OR = 0.25; 95% CI: 0.08–0.77; *P* = 0.0155; Table 3). *Anopheles* spp. habitats located at a distance > 500 m from houses were less likely to contain mosquito pupae than habitats located at a distance of 100–500 m (OR = 0.25; 95% CI: 0.10–0.66; *P* = 0.0050; Table 3). The likelihood of finding mosquito pupae was lower in *Anopheles* spp. habitats surrounded by green areas than habitats surrounded by high-density housing (OR = 0.03; 95% CI: 0.00–0.70; *P* = 0. 0296; Table 3).

## Discussion

As expected, more *Anopheles* spp. breeding habitats were identified in the rainy season than in the dry season. However, mosquito pupae were more likely to be present in *Anopheles* spp. breeding habitats during the dry season than the rainy season. The temperature in Korhogo, which is generally higher during the dry season than in the rainy season, may explain our finding. This higher temperature in the dry season may lead to a shorter larval development time which may reduce the risk of predation [23]. Furthermore, our result may indicate that habitats are more stable in the dry season than the rainy season as a previous study has shown a positive relationship between habitat stability and pupal productivity [24]. It is well known that *Anopheles* spp. breeding habitats are readily flushed out during the rainy season although their numbers and size increase during this period [23]. By contrast, there are fewer larval breeding habitats in the dry season but they are more stable and larval survivorship increases as a result of several factors such as food availability [25]. LSM is also highly effective in the dry season since there are less *Anophele*s spp. breeding habitats to treat, enhancing the feasibility of such strategy [23].

More than 90% of *Anopheles* spp. breeding habitats were surrounded by green areas and the density of *Anopheles* spp. larvae was significantly higher in habitats surrounded by low-density housing and green areas compared to habitats surrounded by high-density housing. Dams, ponds and puddles were more productive of *Anopheles* spp. larvae than rice paddies. One can hypothesize that the oviposition behavior may be different in each type of habitat. Indeed, rice paddies have numerous small pools that are also in close proximity to each other, thus allowing partial oviposition in contrast with dams, ponds and puddles [24]. We found that the latter were, nonetheless, less likely to harbor mosquito pupae than rice paddies. A previous study showed that pupal productivity decreased with increasing larval density [26]. In light of this negative correlation, our results support the hypothesis of higher mortality rate of *Anopheles* spp. larvae in dams, ponds and puddles than in rice paddies. Therefore, rice paddies could be more likely to produce adult malaria vectors than dams, ponds and puddles.

A particularly important result of the present study is the key role of rice paddies in the production of *Anopheles* spp. larvae in the Korhogo area, as they covered a very large surface area (for example, rice paddies amounted to an area of more than 150 ha within a 2 km radius from Nalourgokaha). In a previous study, *Anopheles* spp. larvae were found in more than 60% of the total surface area of rice paddies [27]. In the Korhogo district, rice paddies are generally located at a considerable distance from houses. Numerous studies have shown that female mosquitoes oviposit in habitats in the vicinity of houses for energy conservation purposes [28]. However, unlike in urban areas, gravid vectors have been reported to have a long flight range in rural areas [27]. Furthermore, the density of malaria vectors was very high in the study area [13] and hence the numerous breeding habitats located far away from houses are likely to be the reason for this. This raises the question of where transmission occurs. Indeed, humans are highly mobile, and they spend quite long periods (i.e. several days) in rice-growing areas during the rainy season, allowing malaria transmission at night within the rice-growing areas. Sociological data must complement the ecological data and entomological sampling (both at the larval and adult stages) in order to better understand the spatio-temporal dynamics of malaria transmission in such areas. The entire data set of the REACT project will allow for such an integrated approach in the mid-term future.

In this study, we described the characteristics of *Anopheles* spp. breeding habitats and a striking finding was that over 95% of the sampled *Anopheles* spp. breeding habitats were exposed to sunlight in both seasons. Habitats that are exposed to sunlight are known to be more appropriate for *An. gambiae* (*s.l.*) larvae [29], although the larvae sampled in this study were not identified down to the species level. Adult collection from the same REACT’s project run as a baseline study of malaria transmission confirmed that *Anopheles gambiae* (*s.s.*) was by far the most predominant mosquito species in the Korhogo area, regardless of the season [30]. Unfortunately, this ecological preference, i.e. sunlight exposure, has been shown to negatively impact the efficacy of *Bti* in the field [31]. Indeed, toxins are sensitive to light. Strategies that address this issue will assist with development of the *Bti*-based larvicide strategy for the control of malaria transmission. Ecological studies of immature stages are urgently needed to provide solutions to bypass this issue. For example, treatments carried out in the evening coupled with a frequency adjusted to the recolonization period may help achieve a better immediate performance [31]. The ecological knowledge must be complemented by technological improvements to develop new formulations that result in longer resistance to exposure to sunlight.

We acknowledge a number of methodological limitations of this study. Indeed, despite a concerted effort to be as thorough as possible, we are nonetheless bound to have missed some *Anopheles* spp. breeding habitats. Moreover, due to the numerous water bodies encountered during the rainy season coupled with the large area inspected (a 2 km radius) around each village, only habitats positive for *Anopheles* spp. larvae were described. Therefore, factors associated with the presence/absence of *Anopheles* spp. larvae were not investigated. Consequently, the results must be interpreted with caution.

To further decipher the environmental determinants of malaria vector larval ecology, additional studies that include (i) identification of malaria vector larvae and pupae at the species level; (ii) comparison with human landing catches; and (iii) even phylogenetic studies are needed to better understand the link between larval ecological data and epidemiologically relevant adult vectors. Finally, year-round surveys are needed to obtain more details on the dynamics of *Anopheles* spp. breeding habitats and their productivity in the Korhogo area.

In terms of the ecological data presented in this study, the large surface area of the *Anopheles* spp. breeding habitats in the rainy season has led us to conclude that the period ranging from the end of the rainy season to the dry season is the most appropriate for achieving a cost-effective impact of *Bti*-based larviciding in the Korhogo area. This is consistent with the WHO position on larviciding, which states that LSM is more effective when breeding habitats are limited [8].

## Conclusions

Rice paddies represented the most frequent habitat types for *Anopheles* spp. larvae in the Korhogo area during both the rainy and the dry seasons. *Anopheles* spp. breeding habitats covered large areas during the rainy season, impeding an efficient larviciding activity, whereas they were fewer in number in the dry season. Consequently, we decided to restrict all of the *Bti*-based larviciding efforts to the period ranging from the end of the rainy season to the dry season in this expansive rice-growing area near Korhogo in northern Côte d’Ivoire.

## Abbreviations

LLINs: Long-lasting insecticide nets
*Bti*: *Bacillus thuringiensis israeliensis*
IRS: Indoor residual spraying
ITN: Insecticide treated nets
WHO: World Health Organization
IVM: Integrated Vector Management
LSM: Larval source management
RCT: Randomized controlled trial
IPR: Institut Pierre Richet
